# Secondary headache in emergency

**DOI:** 10.1186/1129-2377-16-S1-A27

**Published:** 2015-09-28

**Authors:** Antonio Carolei, Patrizia Ripa

**Affiliations:** Institute of Neurology, Department of Applied Clinical Sciences and Biotechnology, University of L'Aquila, L'Aquila, Italy

Headache is one of the most common causes of access to Emergency Departments (ED). Primary headaches and headaches secondary to benign conditions (e.g headache attributed to acute sinusitis) represent the majority of cases, while secondary life-threatening headaches are less frequent. The primary objective in the ED setting is to decide whether the headache is primary or secondary and recognize serious life-threatening conditions presenting with headache and requiring prompt medical diagnosis and care, such as subarachnoid hemorrhage (SAH), intracerebral hemorrhage, cerebral venous sinus thrombosis (CVST), cervical artery dissection (CAD), brain tumors, pituitary apoplexy, spontaneous intracranial hypotension, or intracranial infections. Careful history taking and physical examination remain the most important part of the assessment of the headache patient[1]. A thorough history should investigate the onset of headache, quality, location and irradiation of pain, associated symptoms experienced before and during the headache, concomitant medical conditions, medication use, recent trauma or interventions. The examination should then target areas identified as abnormal during the headache history; funduscopy evaluation should be performed when symptoms may suggest an increased intracranial pressure; in addition, a complete neurological assessment including level of consciousness, cranial nerve testing, pupillary responses, motor strength and sensorial testing, and signs of meningeal irritation is essential[2]. Based on historical and physical findings “red flags” for secondary headache disorders are sudden onset, onset after 50 years of age, increased frequency, severity or significant change in the usual headache pattern, new onset with an underlying medical condition (such as cancer or immunodepression), concomitant signs of systemic illness (such as fever, neck stiffness, rash), focal neurologic signs or symptoms, papilledema, and head trauma[2]. In headache patients with one or more “red flags” a diagnostic workup is indicated including blood tests, neuroimaging studies, and cerebrospinal fluid (CSF) examinations, which are selected depending on the patient's history and findings. Blood testing and dosage of inflammation indexes (erythrocyte sedimentation rate, C-reactive protein) should be performed in all headache patients especially when an infective or inflammatory condition is suspected. In the ED, non-contrast computed tomography (CT) is the preferred imaging study and is used to rule out hemorrhage, while most patients should perform a magnetic resonance imaging (MRI) brain scan followed by CT/MRI angiography if brain vessel disease is suspected (such as, CAD, aneurysms, and CVST). Lumbar puncture with CSF analysis may help to diagnose SAH, infection, tumor and disorders related to CSF hypertension or hypotension[3, 4]. Treatment and prognosis depend on the etiology of the headache. Prompt recognition and early treatment of secondary headache are essential to avoid some preventable complications.

## References

[1] Locker TE, Thompson C, Rylance J, Mason SM (2006). The utility of clinical features in patients presenting with nontraumatic headache: an investigation of adult patients attending an emergency department. Headache.

[2] Edlow JA, Panagos PD, Godwin SA, Thomas TL, Decker WW (2008). Clinical policy: critical issues in the evaluation and management of adult patients presenting to the emergency department with acute headache. Annals of Emergency Medicine.

[3] Cortelli P, Cevoli S, Nonino F, Baronciani D, Magrini N, Re G, De Berti G, Manzoni GC, Querzani P, Vandelli A (2004). Multidisciplinary Group for Nontraumatic Headache in the Emergency Department. Evidence-based diagnosis of nontraumatic headache in the Emergency Department: a consensus statement on four clinical scenarios. Headache.

[4] Grimaldi D, Nonino F, Cevoli S, Vandelli A, D'Amico R, Cortelli P (2009). Risk stratification of non-traumatic headache in the emergency department. J Neurol.

